# Simulating Metal Complex Formation and Ligand Exchange: Unraveling the Interplay between Entropy, Kinetics, and Mechanisms on the Chelate Effect

**DOI:** 10.1021/acs.jctc.5c01079

**Published:** 2025-09-11

**Authors:** Luca Sagresti, Luca Benedetti, Kenneth M. Merz, Giuseppe Brancato

**Affiliations:** † 19004 Scuola Normale Superiore, Piazza Dei Cavalieri 7, Pisa I-56126, Italy; ‡ Istituto Nazionale di Fisica Nucleare (INFN), Largo Pontecorvo 3, Pisa I-56127, Italy; § Consorzio Interuniversitario per Lo Sviluppo Dei Sistemi a Grande Interfase (CSGI), Via Della Lastruccia 3, Sesto Fiorentino (Fi) I-50019, Italy; ∥ Department of Chemistry, 3078 Michigan State University, Street, East Lansing 48824, Michigan, United States; ⊥ Department of Biochemistry and Molecular Biology, Michigan State University, Street, East Lansing 48824, Michigan, United States

## Abstract

Metal coordination is ubiquitous in Nature and central in many applications, ranging from nanotechnology to catalysis and environmental chemistry. Complex formation results from the subtle interplay between different thermodynamic, kinetic, and mechanistic contributions, which remain largely elusive to standard experimental methodologies and challenging for typical modeling approaches. Here, considering some prototypical metal complexes between Cd­(II) and Ni­(II) with various amine ligands, we present a comprehensive atomistic-level description of their chemical equilibrium, complex formation, and ligand exchange dynamics in aqueous solution, providing an excellent agreement with available association constants and formation rates spanning several orders of magnitude. This is achieved through an effective molecular simulation approach that combines finely tuned interatomic potentials with state-of-the-art enhanced sampling and kinetics techniques. Worthy of note, the nature of the chelate effect, a fundamental concept in coordination chemistry, is fully unravelled through the comparative analysis of the ligand binding reactions of monodentate and bidentate ligands in octahedral complexes. Results provide a complete picture illustrating all the concurrent contributions to this phenomenon, such as entropy, dissociation rates, and ligand binding mechanisms, in some cases contradicting previously held beliefs. This study represents a step forward for the *in silico* design and applications of coordination complex systems.

## Graphical abstract

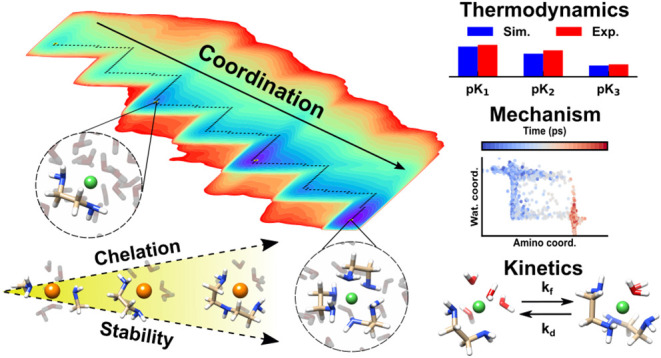

## Introduction

Metal complexes play pivotal roles across diverse domains of chemistry, encompassing catalysis,^1,2^ biochemistry,^3,4^ materials science,^5,6^ and applications in analytical and environmental chemistry.^7^ In recent studies, metal coordination was exploited to design chelating polymers targeting specific transition metals.^8–12^ In molecular biology, metal complexes have been used to probe nucleic acid structures,^13^ to enable site-specific cleavage,^14,15^ and to develop anticancer drugs.^16–18^ In nanotechnology, they have been investigated for their potential application as structural and electron-transfer probes.^19,20^

Nevertheless, recent studies pointed out the importance of a deeper, atomistic-level understanding of metal coordination. Tuning the enthalpy–entropy balance that dictates complex stability is, for example, crucial for improving the catalytic activity and selectivity of organometallic catalysts.^21^ At the same time, unraveling the subtle mechanisms by which metal complexes can activate or hinder fundamental molecular processes allows us to obtain significant breakthroughs in various fields, such as biology^22–24^ and environmental chemistry.^25–27^ Similarly, a deeper comprehension of the chelate effect, one of the most important and widely exploited principles in metal coordination chemistry, would be highly beneficial owing to its widespread applications in various fields.

The chelate effect essentially describes the increased thermodynamic stability of metal complexes formed by multidentate ligands relative to monodentate ligands, provided that the coordination number is the same. Since the seminal work of Schwarzenbach,^28^ many studies have offered simplified models to rationalize the origin of this phenomenon, such as the notion that the (translational) entropy plays a pivotal role in the stability of chelating systems. However, real systems have also shown a non-negligible contribution of the enthalpy,^29^ and a recent study reported enthalpy-driven chelating systems, apparently defying usual convictions.^21^ In another pioneering work, Carter and Beattie^30^ provided a kinetic interpretation of the chelate effect, assuming microscopic reversibility and connecting the association constants with the formation/dissociation rates describing the ligand binding reactions. Since then, unfortunately, kinetic data on complex formation have remained scarce and limited to only a few coordination complexes, such as Pt­(II) and Pd­(II) square-planar complexes and Ni­(II) complexes.^31^ As a consequence, a complete description of the chelate effect that illustrates all its concurrent thermodynamic (i.e., enthalpy–entropy balance), kinetic (i.e., formation/dissociation rates), and mechanistic (i.e., associative vs dissociative ligand binding) contributions is still largely missing.

In this context, *in silico* studies are, in principle, well-suited for gaining atomistic insights into metal binding complexes, especially for structural details.^32–34^ For example, metal ion-ligand interactions can be properly modeled by electronic structure methods^35,36^ or by carefully optimized force fields.^37^ However, the realistic simulation of a metal complex’s equilibrium (i.e., M ⇌ ML ⇌ ML_2_ ⇌···) and ligand exchange dynamics has proven to be far more challenging^38–41^ owing to the long time scale of complex formation and ligand exchange, which are typically >10^–6^ s.^42,43^

Here, a purposely developed *in silico* approach was used to provide, for the first time, a complete picture of the chemical equilibrium and kinetics of metal complexes in solution. The approach combines a recently finely tuned interatomic potential developed by Sengupta et al.,^37^ rooted in the 12-6-4 nonbonded Lennard-Jones potential^44,45^ which proved effective in modeling a range of transition metal assemblies,^46,47^ with enhanced sampling (i.e., metadynamics and its variants^48–50^ ) and kinetics (i.e., Markov State Model^51–53^ ) techniques. As a test case, a series of cadmium (Cd^2+^) and nickel (Ni^2+^) complexes in water with amine ligands of variable denticity and length were considered (Figure 1). The thermodynamic equilibrium between all the stable and metastable ternary species ML_*n*_S_*m*_ (M: metal ion, L: ligand, and S: solvent, with *n*, *m* = 0, 1, ...N) was obtained, showing the relative stability, the energy barriers for interchange, and the minimum free energy pathways leading to complex formation. In addition, mechanistic insights not easily accessible by experiments were also provided, for example, unraveling the nature (i.e., associative or dissociative) of the ligand substitution reaction and the role of the solvent. Predicted stability constants (i.e., *K*_*i*_ = [ML_*i*_]/[ML_*i*–1_]­[L]) and formation/dissociation rates reproduced with great accuracy their experimental counterparts spanning several orders of magnitude. Remarkably, this approach allowed us to shed more light on the origin of the chelate effect in octahedral complexes by quantitatively assessing the thermodynamic, kinetic, and mechanistic contributions underpinning this relevant phenomenon.

**Figure 1:**
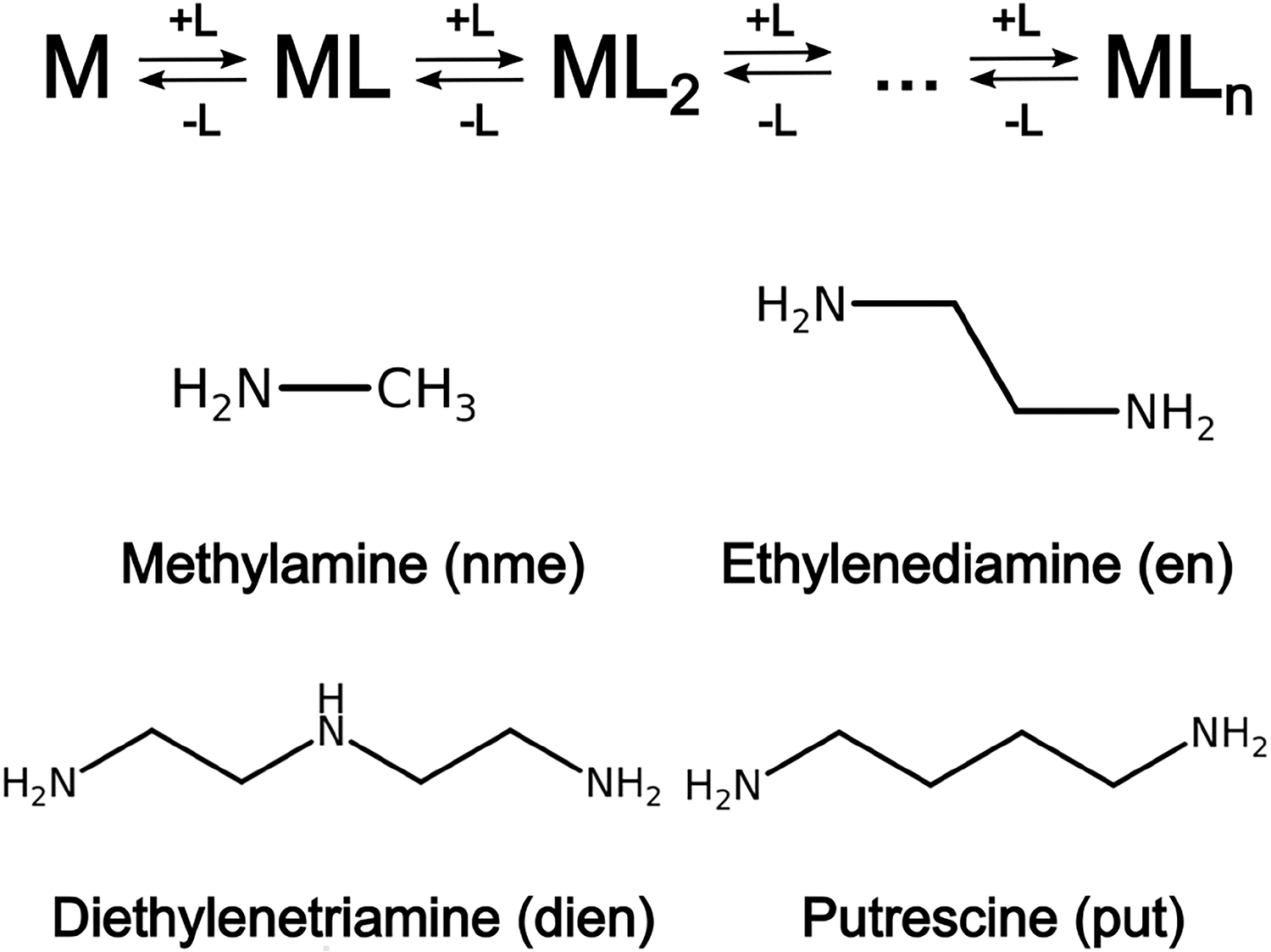
Schematic representation of the various metal–ligand complex species present at equilibrium in aqueous solution and the chemical structures of the amine ligands considered in this work: methylamine (nme), ethylenediamine (en), diethylenetriamine (dien), and putrescine (put). The divalent transition metal ions are Cd­(II) and Ni­(II).

## Methods

### Molecular Dynamics Simulations

Various aqueous solutions of Cd^2+^ (0.05M) and ethylenediamine (en: 0.05 M, 0.1 M, 0.15 M, 0.225 M), methylamine (nme: 0.16 M, 0.30 M), diethylenetriamine (dien: 0.10 M) and putrescine (put: 0.15 M) were considered. To further assess the size consistency of the thermodynamic analysis, two additional Cd­(II)-en systems were prepared with same ratio (1:3), one twice the other. A solution of Ni­(II)-en (en: 0.08 M, ratio 1:3) and Ni­(II)-nme (en: 0.16 M, ratio 1:6) was also prepared to probe the methodology against a system that experimentally has shown slower exchange times. The complete list of systems is shown in Table S1. The 12-6-4 Lennard-Jones type model developed by Li and Merz^44^ was adopted for treating the metal–ligand and metal-water interactions since it was successfully tuned to properly reproduce a range of experimental observables, such as structural properties, hydration free energies, and binding affinities.^45^ In particular, the Cd­(II)-en and Ni­(II)-en interaction model taken from ref 37 was employed in this study and the same metal–nitrogen interaction retained for the dien ligand. On the contrary, the polarizability parameter of the model^37^ was slightly increased (from 3.16 to 3.35, see Table S2) in the case of Cd­(II)-nme (and Cd­(II)-put), to favor the formation of complexes with high coordination numbers (i.e., Cd­(II)­(nme)_4_) as observed experimentally with an excess of monodentate ligand.^54^ The polarizability parameter was also increased (from 2.35 to 2.75) in the case of Ni­(II)-nme, to match the experimental formation constant.^55^ Note that the model overestimates water coordination (8-fold vs 6-fold^56^) around Cd­(II), even though it reproduced accurately the solvation free energy and ion–water distance^44^ and it was fruitfully applied in previous studies.^46,47,57,58^ Despite such a discrepancy, which is reflected in the obtained 2D free energy map, the computed association constants resulted in excellent agreement with experiments, and the description of the substitution reaction (i.e., dissociative mechanism during complex formation) appeared qualitatively correct. The TIP3P^59^ water model was used for modeling the solvent and chloride ions were added to ensure electric charge neutrality of the systems under consideration. Since, in experiments, ClO_4_^–^ and NO_3_^–^ are employed to prevent any significant interaction with the metal and the formation of spurious complexes, we adopted a customized Cd–Cl 12-6 LJ nonbonded model to avoid the formation of ionic couples. Note that experimentally^60^ a stable ionic medium is utilized to keep the activity coefficients of a particular ion constant by introducing a high concentration of a specific anion (e.g., ClO_4_^–^ or NO_3_^–^), which is intended to be unreactive and not form any complexes with the ions under study. By doing so, the activity coefficients of the ions being studied can be considered to remain constant in all the solutions.^61^

All simulations were performed with Amber22 ^62^ enforcing periodic boundary conditions and using the PME^63^ algorithm to treat long-range interactions with a 12 Å, cutoff (10 Å, for the smaller systems).

Minimization was performed using 20000 steps of steepest descent followed by 10,000 steps of conjugate gradient. A 250 ps NPT heating procedure was performed to heat the system from 0 to 300 K followed by a 1 ns equilibration at 300 K with constant *NPT* conditions setting the pressure at 1 atm using Berendsen barostat.^64^ The equilibrated geometries were used for the production runs of 100 ns each. Last frame geometries and coordinates were used as a starting point for the metadynamics simulations (see next paragraph). An integration time step of 1 fs was used in the heating step and 2 fs in the production runs. Langevin dynamics temperature^65^ control was employed in the heating and the production runs with a collision rate equal to 1.0 ps. SHAKE algorithm^66^ was applied to constrain the covalent bonds with hydrogen atoms in the amine ligands in all simulations.

### Thermodynamic Analysis

The stability (or association) constants of metal complexes were obtained directly from the equilibrium populations of the corresponding chemical species, through

1 $$K_{i}=\frac{\left[ML_{i}\right]}{\left[ML_{i-1}\right]\left[L\right]}$$

and, as usual, reported in terms of their logarithm (i.e., p*K*_*i*_ = log* K*_*i*_).^60^

Metal coordination was quantitatively evaluated through a simple and physically sound collective variable originally proposed in ref 67,68 proved effective for studying the water coordination around aqua ions. Accordingly, both water and ligand coordination numbers have been considered, the former based on the Cd^2+^-oxygen (water) distance with a cutoff radius of 3.2 Å (2.9 Å for Ni^2+^), while the latter based on the Cd^2+^-nitrogen (ligand) distance (cutoff radius of 3.4 Å). The well-tempered metadynamics^48,69^ method was used to properly sample the equilibrium population of the various metal–ligand coordination species ([*ML*_*i*_]). Besides, parallel-bias metadynamics (PBMETAD),^70^ together with partition family setup,^71^ was used to bias alternatively one of the metal–ligand/water coordination states. Up to 16 multiple-walkers (MW)^72^ were used to ensure convergence (Cd^2+^ complex systems were simulated for 40 ns each MW with this setup, Ni^2+^ complex system for 80 ns each MW). The deposited Gaussian bias potentials had initial height, width, and deposition stride equal to 1 kJ/mol, 0.1 nm, and 1 ps, respectively; the bias factor was set to 10. Metadynamics simulations were carried out using the open-source, community-developed PLUMED library (ver. 2.8).^73,74^ The free energy surfaces were computed using a reweighting procedure applied to the biased simulation trajectories. Upon convergence, the profiles obtained over the final 10 ns of biased simulations were averaged to determine the reported free energy values Δ*G*_*ij*_, with associated errors as standard deviations.

The minimum free energy pathways of complex formation were evaluated from the corresponding 2D free energy maps using the open-source software MEPSA (ver. 1.6).^75^ The equilibrium population (i.e., concentration) of each complex species was obtained from

2 $$\frac{n_{i}}{n_{j}}=exp^{-\Delta G_{\mathrm{ij}}/k_{B}T}$$

where *n*_*i*_ is the equilibrium population associated with the [*ML*_*i*_] coordination species, Δ*G*_*ij*_ is the difference in free energy between the *i*th and *j*th coordination states, *k*_B_ is the Boltzmann constant and *T* the temperature. From eq 2, it can be shown that the association constants can be expressed in terms of Δ*G*_*ij*_ as

3 $$K_{i}=\frac{\exp\left(-\Delta G_{\mathrm{ij}}/k_{B}T\right)}{\left[L\right]}$$

where the unknown free ligand concentration, [*L*], can be determined from the mass conservation condition:

4 $$\left[L\right]=\left[L_{0}\right]-\sum_{i=1}^{N_{s}}i\left[ML_{i}\right]$$

where [*L*_0_] is the initial ligand concentration and *N*_*s*_ the total number of possible coordination states. Errors relative to p*K*_*i*_ were estimated using a Monte Carlo analysis^76,77^ by repeatedly sampling Δ*G*_*ij*_ from their probability distribution, assuming a normal distribution of these values with standard deviation equal to the error measured by profile average mentioned above. Finally, solving eq 3 to determine the statistical spread of *K*_*i*_. Note that both thermodynamic and kinetic constants depend on the free ligand concentration at equilibrium (eqs 1, 3, and 5). At low ligand-to-metal ratios, the formation of higher-coordination species like ML_3_ becomes statistically rare, leading to poor sampling (see discussion below). In this study, reliable results were obtained from system with a metal–ligand ratio of 1:3. Enthalpies (Δ*H*) of complex formation were obtained from unbiased MD simulations of the bound and unbound state of each metal–ligand complex ([*ML*_*i*_]) (i.e., Δ*H*_*i*_^bind^ = *H*_*i*_^bound^ – *H*_*i*_^unbound^). Binding entropies (Δ*S*) were obtained from the difference between Δ*G* and Δ*H*.

### Markov State Model

The characterization of the configurational space of the metal complexes described by the coordination number allowed the construction of a Markov State Model (MSM) to compute the rate constants between different coordination states. Initial structures were extracted every 0.5 step from the metal-water and metal–ligands coordination maps (≈ 50). From each of these structures 200 unbiased MD replicas were simulated, randomly resampling the momenta. It is important to note that a similar approach using metadynamics to help build a reliable MSM has been used previously to explore the dynamics of the helical peptide Aib9.^78^ Each replica was 2 ns long and the coordination state for ion-ligand and ion–water was recorded every 100 fs. These data were processed using an in-house Python v.3.8 script with the help of the deeptime^79^ python library (see Code Availabilty for the software access). K-Means++^80^ was used to find the lowest number of centers for the MSM that satisfied the implied time scale (see Figure S1) and Chapman-Kolmogorov analysis^52,81^ (see Figure S2). Successively PCCA+^82^ was applied to reduce the MSM microstates (centers) to the actual experimental measurable metal complex states. The transition matrix T_*ij*_ associated with the MSM was also reduced accordingly and mean first passage times (MFPT) were computed using transition path theory (TPT)^83^ and the errors associated were computed using a full Bayesian approach as described in.^81,84^

To compare with experimental rate constants computed MFPT needed to be transformed accordingly taking into account the free ligand concentration. Defining as in the Results section, the kinetic rate *k*_± *i*_ as the association (+*i*) or the dissociation rate (−*i*) for the formation or disruption of the *i*th metal complex *ML*_*i*_, thus from reaction law theory for the *i*th complex state it can be written a kinetic equation of the form

5 $$\frac{d\left[ML_{i}\right]}{dt}=-k_{-i}\left[ML_{i}\right]+k_{i}\left[ML_{\left(i-1\right)}\right]\left[L\right]-k_{i+1}\left[ML_{i}\right]\left[L\right]+k_{-\left(i+1\right)}\left[ML_{i+1}\right]$$

A similar kinetic equation can be written for the populations of the reduced MSM of the form

6 $$\frac{dn_{i}}{dt}=-k_{-i}n_{i}+k_{i}n_{\left(i-1\right)}-k_{i+1}n_{i}+k_{-\left(i+1\right)}n_{i+1}$$

To have compatibility between the experimental and the computed kinetic models, a one to one correspondence must be superimposed. Thus, the forward rate constants of the MSM must be multiplied by a factor γ^2^/*n*_lig_^eq^ where *n*_lig_^eq^ is the number of unbounded ligand at equilibrium (eq 4) and γ = *N*_*Av*_ *V*, with *N*_*Av*_ being the Avogadro number and *V* the volume of the simulation box. Regarding the backward rate constants, the inverse of the MFPT calculated from the MSM must be multiplied just by a factor γ. This correction makes the model robust when dealing with different ion-ligand concentrations as can be seen in Table S3 where we are able to extract very similar kinetic rates for two different ligand concentrations. Note that while equilibrium populations can also be extracted from MSMs using PCCA+, these results depend heavily on the extent to which the underlying unbiased trajectories have sampled relevant states. As an example, the odd intermediate states of the Cd-en system could not be easily characterized kinetically using MSM/PCCA+ owing to the very elusive nature of these configurations. Therefore, we used metadynamics primarily for thermodynamic characterization, ensuring full state-space coverage, and relied on MSM and PCCA+ for the kinetic analysis.

### Mechanism Analysis

To analyze the mechanism of metal complex formation, several unbiased trajectories were generated for 10 Cd^2+^ and 30 en (10 ns each) and 10 Cd^2+^ and 30 nme (5 ns each) systems, saving atomic positions every 0.1 ps. Distances between nitrogen atoms of the amines and Cd­(II) ions were computed. To ignore spurious binding/unbinding events, a ligand was considered bound when its nitrogen atom(s) entered the ion first coordination shell (distance <3.4 Å) and remained for at least 20 ps. The binding events were classified by the change in Cd­(II)-amine coordination (0–1, 1–2, 2–3, 3–4 bindings). The ion-ligand and ion–water coordination numbers were monitored for 20 ps before and after the binding events. The evolution of water/ligand coordination during the binding events was used to classify the ligand exchange mechanism (dissociative and associative). Water-ligand exchange mechanism was also analyzed by monitoring the distances and angles between entering nitrogen, leaving water, and the ion during each binding event, following the work of Falkner et al.^85^

## Results and Discussion

### Metal Complex Equilibrium in Aqueous Solution

An aqueous solution of Cd^2+^ (0.05 M) and ethylenediamine (0.15 M, hereafter referred to as en) at normal conditions was adopted as a prototypical model of metal coordination to illustrate the proposed computational approach. Applications to other amine ligands and/or concentrations are reported below. In analogy with experiments, we ignored the presence of protonated amine species, assuming a moderately basic solution, and we neglected the counterion’s participation in complex formation (see Methods). Metal coordination complexes were described in terms of both water and amino coordination numbers, ML_*n*_S_*m*_ (i.e., for polydentate amines, each amino moiety was counted separately). By performing extended metadynamics simulations, the 2D free energy landscape of the equilibrium solution of the Cd­(II)-en complex was obtained (Figure 2a). The map illustrates the relative stability of all chemical species formed under the given physicochemical conditions and further unravels valuable information about the system, such as the minimum free-energy pathway to complex formation, the interchanging energy barriers, and the nature of the ligand-solvent exchange mechanism (whether associative or dissociative) between different coordination species. Figure 2c shows that [Cd­(en)_2_]^2+^ and [Cd­(en)_3_]^2+^ complexes are the most favorable ones (≈ −30 kJ/mol) with respect to the free metal Cd­(II), as compared to the monocoordinated species, [Cd-en]^2+^ (−17.2 kJ/mol). Figure 2c also shows the peculiar free energy pattern as a function of NH_2_ coordination, highlighting the relative higher stability of even coordination numbers (i.e., n­(NH_2_) = 2, 4, 6) with respect to odd ones (i.e., *n*(NH_2_) = 1, 3, 5). This result clearly shows the bidentate ligand’s preference for chelating ring configurations (i.e., five-membered ring structures).

**Figure 2:**
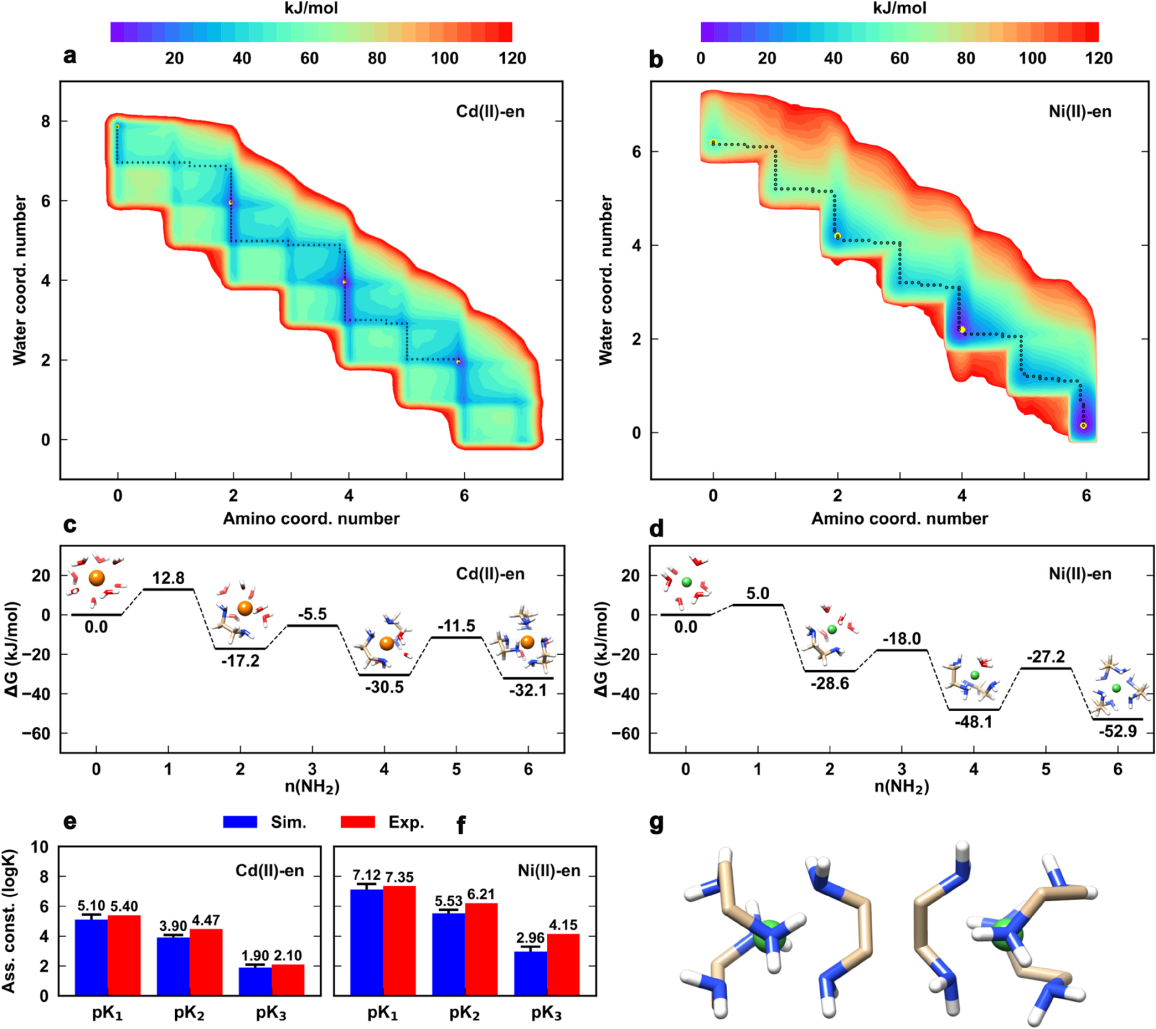
(a) 2D free energy map of the Cd­(II)-en (Cd­(II): 0.05 M, en: 0.15 M), and (b) Ni­(II)-en (Ni­(II): 0.05 M, en: 0.15 M) complex equilibrium in aqueous solution as a function of water and amino coordination number, showing all possible metal coordination states. Yellow points indicate the ML, ML_2_, and ML_3_ configurations. The dotted black lines are the minimum free energy pathways. Note that the profiles depend on the given concentration. (c) Relative stability of the main Cd­(II)-en and (d) Ni­(II)-en complex configurations with respect to the free metal ion. Estimated error is 2 kJ/mol. (e) Computed and experimental association constants (p*K*_*i*_) of the ML, ML_2_, and ML_3_ complex species for the Cd­(II)-en and (f) Ni­(II)-en systems. Estimated errors are reported in Table S6. (g), Representative MD structures of the left-handed (left) and right-handed (right) enantiomers of the Ni­(II)­(en)_3_ complex.

For comparison with experiments, we evaluated the thermodynamic association constants (i.e., *pK*_*i*_, with *i* = 1, 2, 3) from the complex equilibrium population, obtaining a remarkable agreement with the experimental counterparts (Figure 2e). The computational methodology was further assessed by comparing the free energy changes between all the complex species at equilibrium, namely Δ*G*(ML_*i*_ - ML_*i*–1_), as estimated from the experimental *pK*_*i*_ at various Cd­(II)-en concentrations and ratios. Data are reported in Tables S4 and 5. Overall, simulation results showed an excellent agreement with the experimental data (i.e., mean absolute error <1 kcal/mol), with some noticeable deviations observed only in the case of the 1:1 and 1:2 metal–ligand ratio system, for which sampling the ML_3_ population was particularly challenging (Figure S3). Hence, the present approach is robust provided that the ligand is in excess relative to the metal ion (at least three times larger), though a similar limitation is also common in experiments. Note that the equilibrium properties discussed above reflect the remarkable accuracy of the underlying Cd­(II)-en interaction model (a result not necessarily expected since the original force field was optimized toward the interaction of a metal ion with one ligand only^37^). Yet, water coordination was somewhat overemphasized by the model (8-fold vs the usual 6-fold coordination), a result unrelated to the proposed simulation approach and with minor impact on the resulting thermodynamic analysis. In analogy with cadmium, we also investigated the Ni­(II)-en complex, which is known to be characterized by a much higher stability (x100) than Cd­(II)-en. Again, we obtained similar free energy profiles and a favorable agreement with experimental association constants (Figure 2b,d,f). It is worth noting that despite the strong stability of the [Ni­(en)_3_]^2+^ species, both enantiomers (i.e., the left-handed and right-handed propeller illustrated in Figure 2g) were observed to form and disassemble in our simulations.

### Ligand Effects on Complex Stability

The same computational procedure outlined above was extended to investigate methylamine (nme) and diethylenetriamine (dien), to obtain a progressive series of mono-, bi-, and tridentate ligands, and putrescine (put, 1,4-diaminobutane) a bidentate compound with a longer chain than en (Figure 1). For the sake of comparison between the mentioned systems, we considered the same ratio (1:6) between metal ions and ligand amino groups (i.e., 1:6 for Cd­(II)-nme, 1:3 for Cd­(II)-en/put, and 1:2 for Cd­(II)-dien). The computed free energy maps (Figure 3a,b,c) showed an enhanced preference for ligand coordination with increased denticity: at equilibrium, the most favorable complex configurations are those with 2 and 3 bonded NH_2_ for nme, 4 and 6 for en, and 6 for dien. Δ*G*(ML_*n*_) follows a regular pattern for nme and put, in contrast to en and dien where only configurations displaying fully coordinated ligands are stable at equilibrium (Figure 3d–f). As seen for en, Cd­(II)-dien configurations displaying at least one free (not bonded) NH_2_ group were rather unstable. Cd­(II)-put showed an intermediate behavior between nme and en (Figure 3c,f), since the increased chain length and flexibility make this ligand more similar to the monodentate one. Cd­(II)­(put)_2_ was the most stable complex species in solution (−17.2 kJ/mol) at the given concentration, showing comparable stability with the tetra-coordinated Cd­(II)­(nme)_4_ (−14.0 kJ/mol) but rather different than Cd­(II)­(en)_2_ (−30.5 kJ/mol).

**Figure 3:**
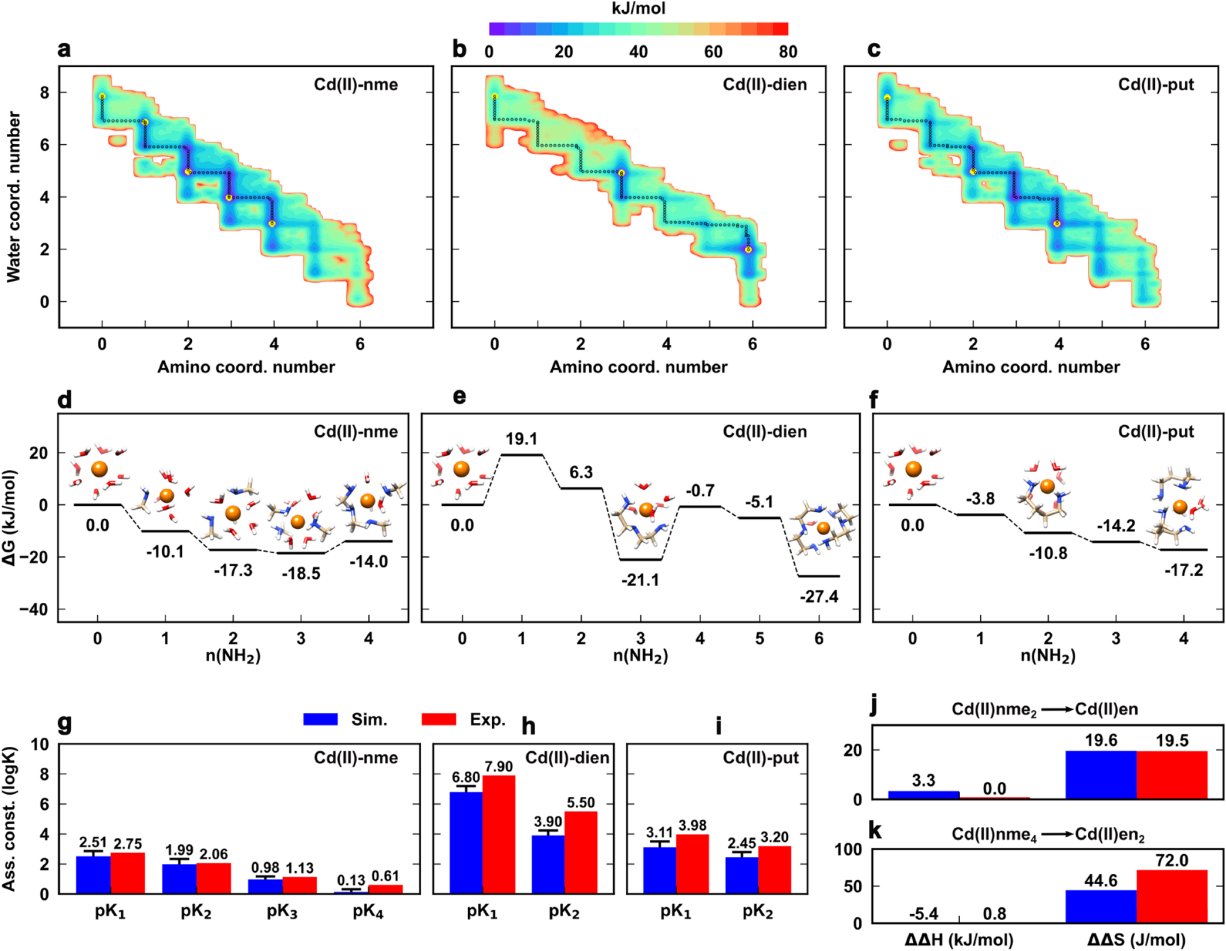
(a) 2D free energy map of the Cd­(II)-nme (Cd­(II): 0.05 M, nme: 0.30 M), (b) Cd­(II)-dien (Cd­(II): 0.05 M, dien: 0.10 M), and (c) Cd­(II)-put (Cd­(II): 0.05 M, put: 0.15) complex equilibrium in aqueous solution as a function of water and amino coordination number, showing all possible metal coordination states. Yellow points indicate the ML, ML_2_, ML_3_, and ML_4_ configurations. The dotted black lines are the minimum free energy pathway. Note that the profiles depend on the given concentration. (d) Relative stability of the main Cd­(II)-nme, (e) Cd­(II)-dien, and (f) Cd­(II)-put complex configurations with respect to the free metal ion. Estimated error is 2 kJ/mol. (g) Computed and experimental association constants (p*K*_*i*_) of the ML, ML_2_, ML_3_, and ML_4_ complex species for the Cd­(II)-nme, (h) Cd­(II)-dien, (i) Cd­(II)-put systems. Estimated errors are reported in Table S6. (j) Computed and experimental enthalpic and entropic energy differences between Cd­(II)­nme_2_ and Cd­(II)­en formation, and (k) between Cd­(II)­nme_4_ and Cd­(II)­en_2_ formation.

*pK*_*i*_’s issuing from simulations and experiments displayed a remarkable agreement (Figure 3g–i). For nme, p*K*_*i*_’s were all very well reproduced, with a slight deviation for p*K*_4_ (about 0.5). Some discrepancies were obtained for put and dien since in these models the metal–ligand interaction potential was borrowed from nme and en, respectively, without further reoptimization (Methods). However, the stability trend provided by the association constants along the amine series was well reproduced: for p*K*_1_, nme < put < en < dien (also, p*K*_1_(en) > pβ_2_(nme) > p*K*_1_(put) and p*K*_1_(dien) > pβ_3_(nme), where β_*i*_ ≡ *K*_1_ *K*_2_...*K*_*i*_). In particular, the chelate effect of en and dien, as defined in the seminal paper by Schwarzenbach,^28^ was in excellent agreement with experiments: p*K*_1_(en) – pβ_2_(nme) = 0.6 (exp: 0.6); p*K*_2_(en) – pβ_4_(nme) + pβ_2_(nme) = 2.73 (exp: 2.79); p*K*_1_(dien) – pβ_3_(nme) = 1.32 (exp: 1.96).

In Table 1, we report the enthalpic (Δ*H*) and entropic (−*T*Δ*S*) contributions to the most relevant metal complex species throughout the amine series, thus allowing a more direct comparison between different systems. It is interesting to note that, when normalized for the number of amino groups, all ligands under scrutiny showed a similar Δ*H* contribution (≈−21 kJ/mol), with the exception of Cd­(II)-dien (≈−17.6 kJ/mol), for the reasons noted above. In contrast, significant differences were observed in the (normalized) entropic contribution: a steady decrease versus ligand denticity was observed in going from nme to dien (i.e., on average 10.9, 8.4, and 5.9 kJ/mol for nme, en, and dien, respectively). ΔΔ*S* for going from Cd­(II)-(nme)_2_ to Cd­(II)-en and from Cd­(II)­(nme)_4_ to Cd­(II)­(en)_2_ were about 20 and 45 J/mol, respectively, in line with experiments and larger than the corresponding enthalpic differences (Figure 3j,k). Overall, results were consistent with previous experiments^42,43,54^ and well illustrated the entropic origin of the chelate effect of bi- and tridentate amine ligands. On the other hand, the enhanced rotational flexibility of put resulted in a rather larger entropic term than the other multidentate ligands (13.8 kJ/mol).

**Table 1:** Thermodynamic Analysis of Cd­(II) Complexes with Various Ligands^a^

ligand_(*i*)_	denticity	Δ*G*	Δ*H*	–*T*Δ*S*	Δ*H*/*n*	–*T*Δ*S*/*n*
nme_1_	1	–14.2	–23.0	8.8	–23.0	8.8
nme_2_	2	–26.8	–46.9	20.1	–23.5	10.1
nme_3_	3	–32.6	–63.2	30.5	–21.1	10.2
nme_4_	4	–33.1	–81.6	48.5	–20.4	12.1
nme_6_	6	–15.9	–98.7	82.8	–16.5	13.8
en_1_	2	–29.3	–43.5	14.2	–21.8	7.1
en_2_	4	–51.9	–87.0	35.1	–21.8	8.8
en_3_	6	–62.7	–119.2	56.4	–19.9	9.4
dien_1_	3	–38.9	–53.4	15.5	–17.8	5.2
dien_2_	6	–61.1	–100.4	39.3	–16.7	6.6
put_1_	2	–18.0	–45.6	27.6	–22.8	13.8
put_2_	4	–31.8	–89.1	57.3	–22.3	14.3

a *n* indicates denticity. Values are in kJ/mol.

### Kinetics of Ligand Binding and Unbinding

Traditionally, kinetic information on complex formation/dissociation reactions is scarce due to limitations of the experimental techniques. In this study, the kinetic analysis of complex formation and ligand exchange was carried out using a Markov State Model (MSM), which allows the evaluation of rate constants spanning several orders of magnitude not readily accessible by standard MD simulations. First, among the several microstates identified by MSM, the PCCA+^82^ analysis identified a few coarse-grained clusters that matched the complex coordination states (i.e., ML_*n*_) previously obtained from the thermodynamic analysis (Figure 4a,b). Then, the mean first passage times among the different states were evaluated along with the corresponding rate constants. Figure 4c–e report the computed rate constants for all complex association/dissociation reactions concerning Ni­(II)-en, Ni­(II)-nme and Cd­(II)-en.

**Figure 4:**
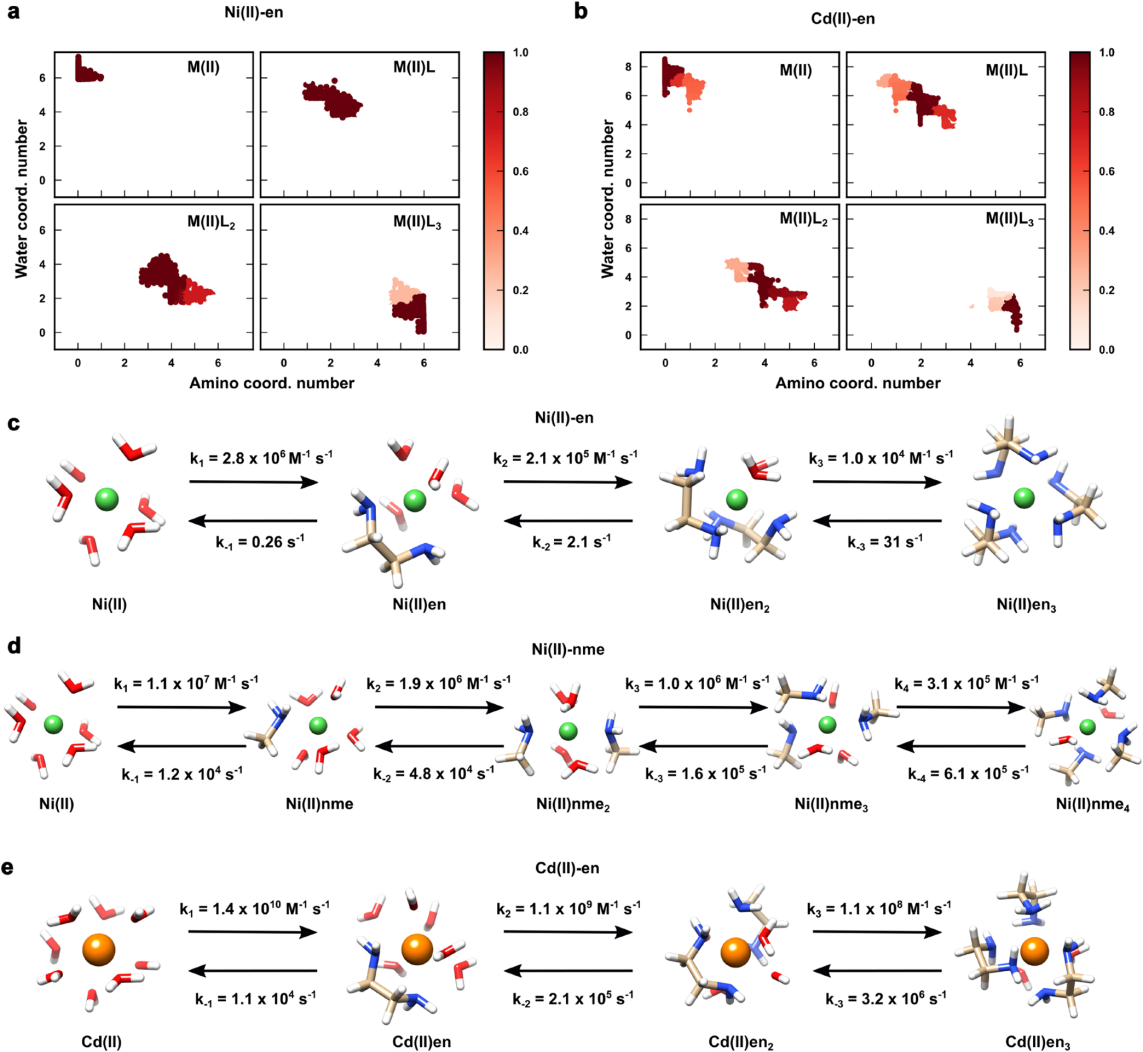
(a) Macrostate assignment probability of the short unbiased MD trajectories following PCCA+ analysis for Ni­(II)-en and (b) Cd­(II)-en. The four macrostates identified by the algorithm accurately correspond to distinct coordination numbers (ML_*i*_) of the metal ion with the ethylenediamine. (c) Computed formation (*k*_*i*_) and dissociation (*k*_–*i*_) rate constants for Ni­(II)-en, (d) Ni­(II)-nme, (e) and Cd­(II)-en. Estimated errors are reported in Table S7. 2D free energy map, relative stability of the main complexes and association constant (p*K*_*i*_) of Ni­(II)-nme system are shown in Figure S4.

We observed that Ni­(II)-en complex formation (i.e., forward) rates were consistently greater than the corresponding dissociation (i.e., backward) rates, with a ratio of *k*_*i*_/*k*_–*i*_ spanning a range from 10^3^ to 10^7^. The backward reactions were particularly slow in agreement with the stability of Ni­(II)-en complex species. Remarkably, experimental measurements obtained from stopped-flow techniques on Ni­(II)-en complex solutions reported data in very good agreement with the present predictions. We obtained *k*_1_ = 2.8 × 10^6^ M^–1^ s^–1^ and *k*_–1_ = 0.26 s^–1^ for the first ligand binding and unbinding event, as compared to the experimental rates (*k*_1_ = 3.5 × 10^5^ M^–1^ s^–1^ and *k*_–1_ = 0.08 s^–1^).^86^ Also, an excellent agreement was found for the third ligand association/dissociation rates, where we found *k*_3_ = 1.0 × 10^4^ M^–1^ s^–1^ (exp: 1.1 × 10^4^ M^–1^ s^–1^) and *k*_–3_ = 31 s^–1^ (exp: 38 s^–1^ ^87^). Besides, it is worth noting that both experiments and simulations displayed similar forward reaction (*k*_1_) and water exchange rates (*k* ^H_2_O^ = 1.8 × 10^5^ s^–1^, exp: 3.37 × 10^4^ s^–1^ ^88^), supporting the view that the release of a water molecule from the first coordination shell is the rate-determining step for complex formation (i.e., dissociation mechanism).^89,90^ This was further corroborated by results obtained for Cd­(II)-en (*k*_1_ = 1.4 × 10^10^ M^–1^ s^–1^ vs *k* ^H_2_O^ *=* 5 × 10^10^ M^–1^ s^–1^), though showing a much faster kinetics (about × 10^4^) than Ni­(II) (Figure 4e).

Note that stability constants (p*K*_*i*_) evaluated from the *k*_*i*_/*k*_–*i*_ ratio aligned fairly well with those obtained from the previous thermodynamic analysis (Table S8) and similar rate constants were obtained when testing different metal ion-ligand concentrations (Table S3), as further proof of the robustness of our model.

### Water-Ligand Exchange Mechanisms

Metal complexes in aqueous solutions are formed through a substitution reaction between water and the ligand in the first ion coordination shell,^91^ where according to Langford and Stengle’s classification^92^ such an exchange follows either an associative or a dissociative mechanism. Previous experimental studies on polyamine complexes with transition metal ions supported a dissociative mechanism, starting from the pioneering work of Eigen and co-workers.^89,93^ In particular, kinetic studies hinted that the loss of a water molecule from the first coordination shell represents the rate-determining step in metal complex formation,^55,89,94,95^ based on the close agreement between the water exchange and the first-ligand binding rates.^89,96^

For all Cd­(II) systems under scrutiny, we observed that the release of a coordinating solvent molecule into the bulk solution preceded the first ligand binding event, as shown by the minimum free energy pathway of complex formation (Figures 2a and 3a–c), though the fine details were different, as discussed in the following. We analyzed in some detail the binding mechanism of ethylenediamine to a free Cd^2+^ from multiple unbiased simulations by closely following the coordination shell around the metal ion for a few picoseconds before and after the binding event (Methods). The coordination of the bidentate ligand occurred as a two-step reaction: first, we observed the binding of one NH_2_ group to the metal ion (Figure 5a), then the second amino moiety entered within the first coordination shell of Cd^2+^ (Figure 5a). Monitoring the evolution of the coordinating water molecules around the metal center, we observed a dissociative mechanism upon first NH_2_ binding, with a water molecule leaving the Cd^2+^ coordination shell before the ligand (Figure 5a). However, the second amino binding event, leading to a fully coordinated bidentate ligand, followed either an associative or a dissociative mechanism (Figure 5b). The associative mechanism led to a temporary overcoordination of Cd^2+^ during the en chelating ring formation. Similar considerations apply when a second en molecule binds to Cd^2+^ (Figure S5). On the other hand, Figure 5c shows that only a dissociative mechanism is feasible upon the second amino binding event when considering nme as a ligand (i.e., Cd­(II)-nme → Cd­(II)­(nme)_2_), thus highlighting another difference between polydentate ligands versus monodentate ones. In particular, the discovery of an associative mechanism upon chelate formation (i.e., the ring closure step) has important consequences on the kinetics of this event (*vide infra*).

**Figure 5:**
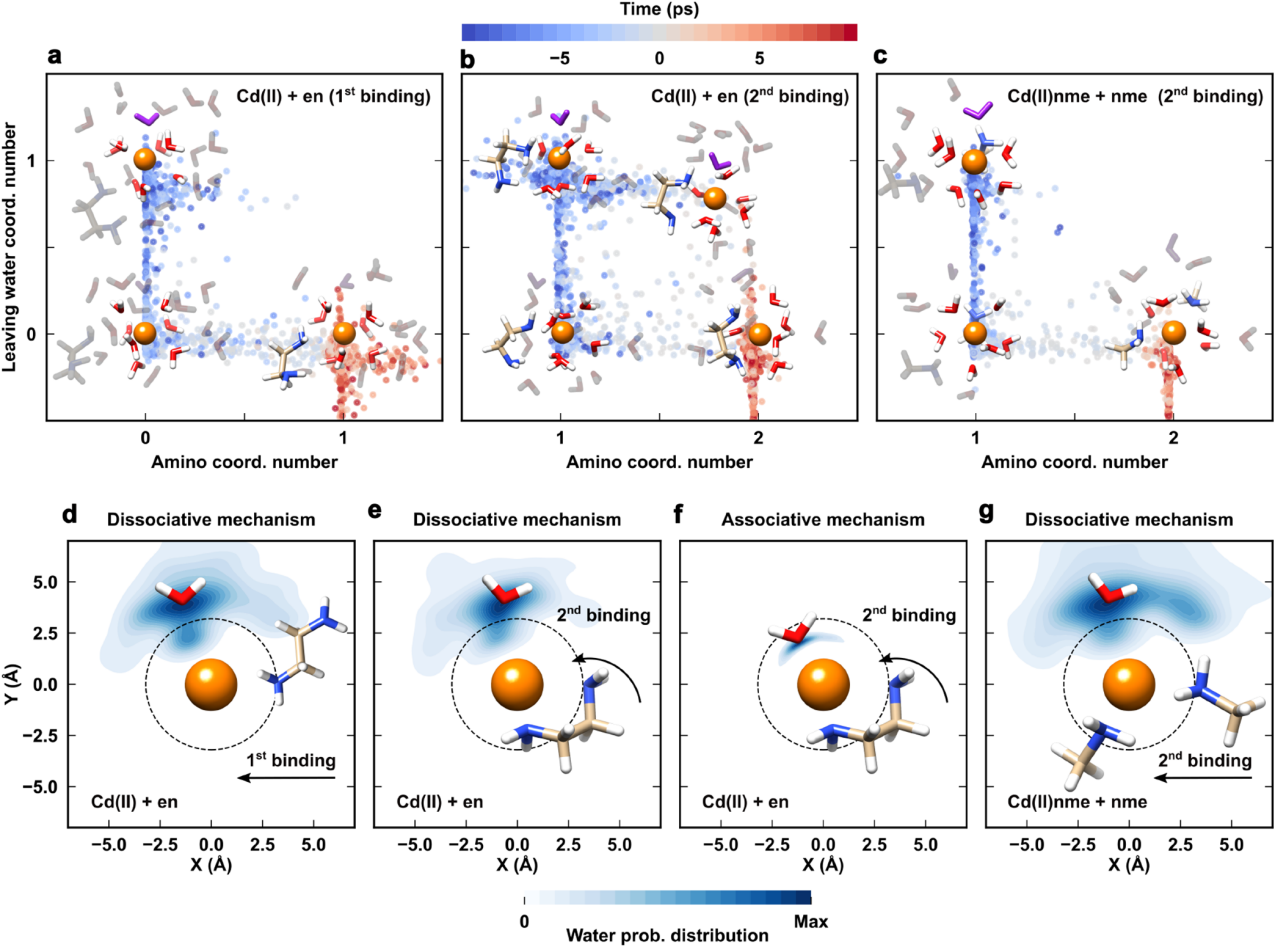
(a) Evolution of amino and leaving water coordination number in the first and (b) second binding event of Cd­(II)­en formation, and (c) in the second binding event of Cd­(II)­nme_2_ formation. The leaving water is represented in purple. (d) Probability distribution of the leaving water in the time interval from 5 ps before to 5 ps after the first dissociative binding, (e) the second dissociative and (f) second associative binding events of Cd­(II)­en formation, and (g) the second dissociative binding event of Cd­(II)­nme_2_ formation. The black dotted circle represents the Cd­(II) first solvation shell.

Furthermore, we analyzed the relative orientation of the leaving water molecule with respect to Cd^2+^ and the entering (NH_2_) coordination group by projecting the coordinates of Cd^2+^, H_2_O and NH_2_ on the XY plane, while keeping the metal ion at the origin and the amino group along the *X*-axis. The analysis focused on the distribution of the leaving water molecule during the first and second binding event of Cd­(II)-en formation and the second binding event of Cd­(II)­(nme)_2_. In all events, the leaving H_2_O exited from the coordination shell along a direction orthogonal (about 90°) to the entering amino moiety (Figure 5d–g). The only noticeable difference was that in the Cd­(II)-en associative mechanism the water molecule resided inside the first coordination shell when the NH_2_ binding occurred (Figure 5f).

### Insights into the Chelate Effect in Octahedral Metal Complexes

Despite being one of the most well-established concepts in coordination chemistry, the subtle interplay between thermodynamic, kinetic, and mechanistic aspects that gives rise to the chelate effect in metal complexes remains largely unknown. Considering the reactions between a bidentate ligand and a metal center, we have
7

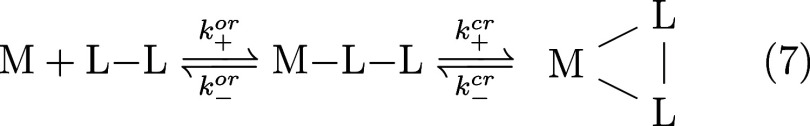

8 $$K^{or}=\frac{k_{+}^{or}}{k_{-}^{or}}K^{cr}=\frac{k_{+}^{cr}}{k_{-}^{cr}}$$

where the intermediate stability constants (*K* ^or^, *K* ^cr^) relative to the first (“open ring”) and second (“closed ring”) semireactions are related to the ligand binding/unbinding rate constants (*k*_+_^or/cr^, *k*_–_^or/cr^), assuming microscopic reversibility. Note also that *K*_1_^L–L^ ≡ *K* ^or^ *K* ^cr^ where *K*_1_^L–L^ is the first association constant of the bidentate ligand. Moreover, the overall formation (*k*_*f*_) and dissociation (*k*_*d*_) rate constants are related to the open/closed ring rate constants according to

9 $$k_{f}=\frac{k_{+}^{\mathrm{or}}k_{+}^{\mathrm{cr}}}{k_{-}^{\mathrm{or}}+k_{+}^{\mathrm{cr}}}k_{d}=\frac{k_{-}^{\mathrm{or}}k_{-}^{\mathrm{cr}}}{k_{-}^{\mathrm{or}}+k_{+}^{\mathrm{cr}}}$$

It is worth noting that while there exists overwhelming evidence of the extra stability of metal complexes formed by bidentate (or polydentate) ligands with respect to monodentate ones (*K*_1_^L–L^ > *K*_1_^L^ *K*_2_^L^, with L and L–L monodentate and bidentate ligand, respectively), the relative contribution of the first and second binding event (eq 8) remains unknown. This is mainly due to the lack of experimental characterization of the intermediate species, i.e., the so-called open-ring configurations during bidentate ligand binding, for which kinetic and/or thermodynamic information is scarce, if any. One notable exception is the study by Carter and Beattie^30^ on square-planar platinum complexes, which, however, provided only partial information on the relevant rate constants, i.e., the ring closure rates (*k*_+_^cr^). Based on such an incomplete experimental characterization, it was argued that in octahedral complexes both the forward (*k*_*f*_) and the reverse (*k*_*d*_) reaction rates are determined by the rates of dissociation, in the forward reaction that of water (see discussion above) and in the reverse reaction that of the amine group (*k*_–_^cr^); besides, it was conjectured that the thermodynamic stability of the open ring configuration was already greater than the corresponding monodentate configuration, in other words, it was predicted *K* ^or^ > *K*_1_^L^.^30^

Our thermodynamic analysis unraveled that not only the open ring configuration is much less stable than the closed ring configuration (*K* ^or^ ≪ *K* ^cr^) for both Cd­(II)-en and Ni­(II)-en, but also less stable than the one monodentate configuration (*K* ^or^ < *K*_1_^L^, with *K*_1_^L^ the first association constant of the nme ligand), a result that contradicts some common beliefs, though unsupported by experiments. For both Cd­(II)-en and Ni­(II)-en complexes, log *K* ^or^ is about 0.6, while log *K*_1_(nme) ≥ 2.1 (Table 2). For comparison, we also considered Cd­(II)-put, which also reported a less favorable intermediate species (singly bound ligand) than Cd­(II)-nme, though more stable than Cd­(II)-en (log *K* ^or^(put) = 1.9).

**Table 2:** Association Constants and Formation/Dissociation Rate Constants of the Cd­(II)-en, Ni­(II)-en and Cd­(II)-put Complexes

association const.	Cd(II)-en	Ni(II)-en	Cd(II)-put
log *K* ^or^	0.6	0.7	1.9
log *K* ^cr^	4.5	6.4	1.2
log *K* ^or^ *K* ^cr^ ≡ p*K*_1_	5.1	7.1	3.1

rate constants	Cd(II)-en	Ni(II)-en	
*k*_+_^or^	2.9 × 10^10^ M^–1^ s^–1^	2.8 × 10^6^ M^–1^ s^–1^	
*k*_–_^or^	7.3 × 10^9^ s^–1^	5.6 × 10^5^ s^–1^	
*k*_+_^cr^	6.8 × 10^9^ s^–1^	7.2 × 10^9^ s^–1^	
*k*_–_^cr^	2.1 × 10^5^ s^–1^	2.9 × 10^3^ s^–1^	
*k*_*f*_	1.4 × 10^10^ M^–1^ s^–1^	2.8 × 10^6^ M^–1^ s^–1^	
*k*_*d*_	1.1 × 10^4^ s^–1^	2.6 × 10^–1^ s^–1^	

As noted previously, the chelate effect between Cd­(II)-en and Cd­(II)­(nme)_2_ is mostly due to an entropic gain (ΔΔ*S* = 19.6 J/mol). However, by decomposing the contribution issuing from the first and second amino binding event, we obtained that ΔΔ*S*_1_ = −31.8 J/mol and ΔΔ*S*_2_ = 51.5 J/mol, meaning that the entropic advantage of the bidentate ligand is provided solely by the ring closure step of the complex reaction, in contrast the open ring configuration is entropically unfavorable with respect to monodentate binding. To provide a molecular interpretation of the negative ΔΔ*S*_1_, we have analyzed the change in the hydrogen bonding pattern and in the rotational flexibility of en in going from the unbound to the singly bound configuration, as compared to nme binding. The decrease in the number of hydrogen bonds with water was similar between en and nme (≈0.3, see Table S9), hence the release of water molecules did not account for the entropy difference. The torsional motion of en, in contrast, appeared more restricted when this ligand is singly bound to the central metal (Figure S6), a result well in line with ΔΔ*S*_1_ < 0. Interestingly, the initial amino group binding of put was comparatively less unfavorable than en with respect to the monodetantate ligand (ΔΔ*S*_1_ = −10.0 J/mol), due to a less significant hindering of the intramolecular rotational motion in the open ring configuration. Nevertheless, in the case of putrescine, the chelate effect entirely vanished upon ring formation (Cd­(II)-put, log *K*_1_ = 3.1; Cd­(II)-nme, log β_2_ = 4.5), a result fully supported by experiment (Figure 3g,i).

As a further investigation of the chelate effect, we focused on the kinetic analysis of en ligand binding/unbinding to both Cd­(II) and Ni­(II). As reported in Figure 4c,d and Table S7, the backward (dissociation) reactions ML → M for the monodentate (nme) ligand were 2 to 5 orders of magnitude greater than the corresponding one for en, while the forward (formation) reaction rates were about the same. This finding was consistent with the notion that the enhanced stability of metal complexes with chelating agents is mostly due to slower dissociation rates.^90^ In this case, the common belief is that it is the first amino group detachment (*k*_–_^cr^) of the bound bidentate ligand to be the rate-determining step in the dissociation process. However, a more detailed analysis highlighted some important differences between the Cd­(II)-en and the Ni­(II)-en complex, as summarized in Table 2. Evaluation of the ring closure step (*k*_+_^cr^) reported similar and relatively fast rate constants (Cd­(II), 6.8 × 10^9^ s^–1^; Ni­(II), 7.2 × 10^9^ s^–1^) in line with an intramolecular (associative) mechanism not dependent on the solvent role, as observed in preceding section. Moreover, the overall formation rate constants (*k*_*f*_) appeared to be essentially determined by the first amino binding, that is *k*_*f*_ ≈ *k*_+_^or^, despite the complex formation rate was rather different in the two cases reflecting the relative water exchange rates (as discussed above).

On the other hand, the dissociation rate constants were not only quantitatively different, but they appeared to be dependent on distinct contributions of the two-step process described above. For Cd­(II)-en, we found that *k*_*d*_ ≈ *k*_–_^cr^, hence the ligand dissociation rate was essentially determined by the chelate ring breaking. Note how, in this case, *k*_–_^*or*^ and *k*_+_^cr^ were comparable (∼10^9^ s^–1^), as reported in Table 2. In contrast, for Ni­(II)-en the dissociation rate was about 10^4^ times slower than the corresponding chelate ring opening (*k*_*d*_ = 2.6 × 10^–1^ s^–1^, *k*_–_^cr^ = 2.9 × 10^3^ s^–1^). In fact, *k*_+_^cr^ was much larger than *k*_–_^or^ and, as a result, the observed kinetic inertia to ligand unbinding was due to the combined effect of both “fast” ring closure (*k*_+_^cr^) and “slow” ring breaking (*k*_–_^cr^), which also, in turn, determined the observed extra stability of the fully bound ligand configuration, log (*k*_+_^cr^/*k*_–_^cr^) = 6.4.

These results support the view that the chelate effect is entirely determined by the ring closure step (*K* ^cr^ = *k*_+_^cr^/*k*_–_^cr^) from both a thermodynamic and a kinetic standpoint. This finding is consistent with what was found for square-planar complexes, but contradicts previous predictions on octahedral complexes.^30^ On the other hand, the open ring configuration formed by a singly bound bidentate ligand is less favorable than the corresponding monodentate ligand complex, since the intramolecular flexibility of the former becomes more restricted in the bound state. Moreover, a kinetic interpretation of the closed ring increased stability (*K* ^cr^ > *K*_2_^L^) is proposed, which is based on the observation of a relatively “fast” ring formation (*k*_+_^cr^) enabled by an associative mechanism not dependent on the nature of the metal or on the solvent dissociation, and on the “slow” ring opening step (*k*_–_^cr^), which is affected by steric restriction during bidentate twisting not occurring in the case of the monodentate ligand release (the latter was estimated to be at least 10× faster^97^). Note also that *k*_–_^cr^ is modulated by the specific metal–ligand interaction and, as a consequence, it accounts for the observed stability increase of Ni­(II)-en complexes relative to Cd­(II)-en (K_1_(Ni/Cd) ≈ 75) of the closed ring configuration (Table 2).

## Conclusions

In this work, we presented a comprehensive molecular view of metal complex equilibrium in water obtained using a simulation approach based on advanced enhanced sampling and MSM methodologies. This approach makes the detailed thermodynamic and kinetic analysis of metal coordination systems computationally feasible, thus allowing direct comparison with experiments. Results obtained on a series of metal amine complexes showed an agreement with observed stability constants (p*K*_*i*_) and relative free energies (Δ*G*(ML_*i*_)) within chemical accuracy (1 kcal/mol). Complex formation and ligand exchange rates reproduced available experimental results, thus supporting application to a large number of systems for which kinetic information is presently lacking. Noteworthy, simulations of metal-amine complexes with variable ligand denticities allowed us to fully underscore the nature of the chelate effect as characterized by the interplay between entropic contributions, dissociation rates, and ligand binding mechanisms. Results show that some details of the chelate effect are rather system-specific, such as the relative contribution of the intermediate metal–ligand configurations with respect to the fully bound ones. Therefore, it remains to be seen if other classes of ligands (e.g., carboxylates) share similar features with the amines. We believe that the demonstrated ability to gain valuable insights into metal complex formation and ligand exchange makes the present approach well-suited for computer-aided design in coordination chemistry and a useful tool for better understanding natural processes triggered or modulated by metal binding.

## Supplementary Material



## Data Availability

Input files for reproducing MD simulations and enhanced sampling simulations described in the Methods section, together with a pipeline to reweight the results are available at the Zenodo repository at the link https://zenodo.org/records/15230404. The code to compute equilibrium constants from free energy profiles issuing from enhanced sampling is available at https://github.com/SNS-Brancato-Lab/metals-ligand-equilibrium.git. The code to perform the analysis of ligand-exchange mechanism is available at https://github.com/SNS-Brancato-Lab/mechanism_ligand_exchange.git. The code that performs MSM calculation, evaluation and kinetic analysis is available at https://github.com/SNS-Brancato-Lab/MSManalysis.git

## Notes

Additional simulation details, complete list of computed stability and rate constants, hydrogen bond and dihedral angle analysis of ligands during complex formation, implied time scale and Chapman-Kolmogorov tests (PDF)

## #

Departement of Physics, Freie Universität Berlin, Arnimallee 12, 14195 Berlin, Germany
