# Supplementary material for: Simulating Metal Complex Formation and Ligand Exchange: Unraveling the Interplay between Entropy, Kinetics, and Mechanisms on the Chelate Effect
Source: J Chem Theory Comput. 2025 Sep 11;21(18):8950–62. doi: 10.1021/acs.jctc.5c01079 (PMC12461782; doi:10.1021/acs.jctc.5c01079)
Supplement: Supplementary file 1 [file ct5c01079_si_001.pdf]

Supplementary Information for: Simulating Metal Complex Formation and Ligand Exchange: Unraveling the Interplay Between Entropy, Kinetics, and Mechanisms on the Chelate Effect

Luca Sagresti,[†,‡,¶,#] Luca Benedetti,[†,‡,#] Kenneth M. Merz Jr.,[§,‖] and Giuseppe Brancato[*,†,‡,¶]

†*Scuola Normale Superiore, Piazza Dei Cavalieri 7, Pisa, I-56126, Italy*

‡*Istituto Nazionale di Fisica Nucleare (INFN), Largo Pontecorvo 3, Pisa, I-56127, Italy*

¶*Consorzio Interuniversitario per Lo Sviluppo Dei Sistemi a Grande Interfase (CSGI), Via Della Lastruccia 3, Sesto Fiorentino (Fi), I-50019, Italy*

§*Department of Chemistry, Michigan State University, Street, East Lansing, 48824, Michigan, United States*

‖*Department of Biochemistry and Molecular Biology, Michigan State University, Street, East Lansing, 48824, Michigan, United States*

⊥*Present address: Departement of Physics, Freie Universität Berlin, Arnimallee 12, 14195 Berlin, Germany*

#*L.S. and L.B. contributed equally to this work*

E-mail: giuseppe.brancato@sns.it

Contents

List of Tables

1 Simulation details 3
2 Cd(II)-nme stability constants using different polarizability parameters . . . 4
3 Cd(II)-en formation and dissociation rate constants at different concentrations 5
4 Cd(II)-en stability constants at different concentrations 6
5 Cd(II)-en free energy changes at different concentrations 7
6 Experimental and computed stability constants 8
7 Computed formation and dissociation rate constants 9
8 Stability constants evaluated from formation and dissociation kinetics 10
9 Hydrogen bonding analysis of metal-amine complex formation 11

List of Figures

1 Cd(II)-en implied timescale plot 12
2 Chapman-Kolmogorov test 13
3 Relative stability of Cd(II)-en complexes at different concentrations 14
4 Thermodynamics of Ni(II)-nme complexes in aqueous solution 15
5 Water-ligand exchange mechanisms in Cd(en)$_2$ complex formation 16
6 Ethylenediamine and putrescine dihedral angle evolution during chelation. . 17

Table S 1: Simulation details of all systems considered in this work.

System	#Metals	#Ligands	Volume(nm^3)	Ligand Conc.(M)
Cd-en	1	3	64	0.08
Cd-en	2	6	128	0.08
Cd-en	10	10	330	0.05
Cd-en	10	20	330	0.10
Cd-en	10	30	330	0.15
Cd-en	10	45	330	0.22
Cd-nme	1	6	64	0.16
Cd-nme	10	60	330	0.30
Cd-dien	10	20	330	0.10
Cd-put	10	30	330	0.15
Ni-en	1	3	64	0.08
Ni-nme	1	6	64	0.16

Table S 2: Experimental and computed stability constants (pK_i) of the Cd(II)-nme complex, using different polarizability values for the 12-6-4 LJ model between Cd(II) and nme. See details in the Method section.

	pK_1	pK_2	pK_3	pK_4
exp[a]	2.75	2.06	1.13	0.61
α_{Cd-nme}=3.35[b]	2.51	1.99	0.98	0.13
α_{Cd-nme}=3.16[c]	2.10	1.21	0.22	-0.81

[a] Ref.[1]

[b] Polarizability parameter used in this work.

[c] Polarizability parameter from ref.[2].

Table S 3: Computed formation and dissociation rate constants (k_i) of the Cd(II)-en complex, evaluated between different metal-ligand coordination states and at two different concentrations, 0.08 M and 0.15 M.

Ligand conc.	k_1 $(M^{-1}s^{-1})$	k_2 $(M^{-1}s^{-1})$	k_3 $(M^{-1}s^{-1})$
0.08 M	$4.1 \pm 1.1 \cdot 10^{10}$	$6.6 \pm 0.1 \cdot 10^{9}$	$3.89 \pm 0.08 \cdot 10^{8}$
0.15 M	$1.4 \pm 0.2 \cdot 10^{10}$	$1.14 \pm 0.02 \cdot 10^{9}$	$1.09 \pm 0.02 \cdot 10^{8}$
	k_{-3} (s^{-1})	k_{-2} (s^{-1})	k_{-1} (s^{-1})
0.08 M	$3.17 \pm 0.07 \cdot 10^{6}$	$4.2 \pm 0.7 \cdot 10^{5}$	$3.5 \pm 0.6 \cdot 10^{4}$
0.15 M	$3.16 \pm 0.06 \cdot 10^{6}$	$2.05 \pm 0.04 \cdot 10^{5}$	$1.1 \pm 0.1 \cdot 10^{4}$

Table S 4: Experimental and computed stability constants (pK_i) of the Cd(II)-en complex as evaluated at different system concentrations

Cd(II)-en	pK_1	pK_2	pK_3
exp[a]	5.4	4.47	2.1
1-3	5.2 ± 0.4	4.0 ± 0.2	1.8 ± 0.3
2-6	5.1 ± 0.4	4.1 ± 0.2	1.8 ± 0.3
10-45	5.1 ± 0.4	3.8 ± 0.2	1.7 ± 0.2
10-30	5.1 ± 0.3	3.9 ± 0.2	1.9 ± 0.2

[a] Ref.[3]

Table S 5: Free energy changes (ΔG_{ij}) of the Cd(II)-en complex evaluated considering different metal-ligand coordination states at different concentrations. In parentheses are the values predicted from the experimental stability constants at the same concentrations. See the Methods section for details.

Cd(II)-en	ΔG_{01} (kJ/mol)	ΔG_{12} (kJ/mol)	ΔG_{23} kJ/mol
1-3	18.5 (19.5)	12.8 (14.2)	-0.3 (1.3)
2-6	18.4 (19.4)	12.7 (14.1)	-0.3 (1.2)
10-45	23.7 (24.9)	15.6 (19.5)	3.6 (6.7)
10-30	17.2 (20.4)	13.3 (15.1)	1.6 (2.4)
10-20	15.4 (12.9)	3.8 (7.0)	-5.2 (-7.4)
10-10	5.0 (2.7)	-5.2 (-2.7)	-26.1 (-15.5)
**MAE**	1.9	2.4	3.9

Table S 6: Stability constants (pK_i) relative to different metal-ligand coordination states for all systems under investigation. Available experimental data are reported in parentheses.

M-L	pK_1	pK_2	pK_3	pK_4
Cd(II)-en	5.10 ± 0.35<br>(5.40)[a]	3.90 ± 0.18<br>(4.47)[a]	1.90 ± 0.19<br>(2.10)[a]	
Cd(II)-nme	2.51 ± 0.35<br>(2.75)[b]	1.99 ± 0.35<br>(2.06)[b]	0.98 ± 0.19<br>(1.13)[b]	0.13 ± 0.19<br>(0.61)[b]
Cd(II)-dien	6.80 ± 0.39<br>(7.90)[c]	3.90 ± 0.33<br>(5.50)[c]		
Cd(II)-put	3.11 ± 0.40<br>(3.98)[c]	2.45 ± 0.34<br>(3.20)[c]		
Ni(II)-en	7.12 ± 0.38<br>(7.35)[d]	5.53 ± 0.23<br>(6.21)[d]	2.96 ± 0.33<br>(4.15)[d]	
Ni(II)-nme	2.16 ± 0.37<br>(2.23)[e]	1.48± 0.19	0.55 ± 0.19	-0.86 ± 0.53

[a] Ref.[3]
[b] Ref.[1]
[c] Ref.[4]
[d] Ref.[5]
[e] Ref.[6]

Table S 7: Computed formation and dissociation rate constants (k_i) between different metal-ligand coordination states, considering the Cd(II) and Ni(II) complexes with both nme and en.

ML	k_1 $(M^{-1}s^{-1})$	k_2 $(M^{-1}s^{-1})$	k_3 $(M^{-1}s^{-1})$	k_4 $(M^{-1}s^{-1})$
Cd(II)-en	$1.4 \pm 0.2 \cdot 10^{10}$	$1.1 \pm 0.1 \cdot 10^{9}$	$1.1 \pm 0.1 \cdot 10^{8}$	
Cd(II)-nme	$1.2 \pm 0.1 \cdot 10^{10}$	$1.3 \pm 0.1 \cdot 10^{10}$	$6.5 \pm 0.1 \cdot 10^{9}$	$1.4 \pm 0.1 \cdot 10^{9}$
Ni(II)-en	$2.8 \pm 0.3 \cdot 10^{6}$	$2.1 \pm 0.3 \cdot 10^{5}$	$1.0 \pm 0.1 \cdot 10^{4}$	
Ni(II)-nme	$1.1 \pm 0.1 \cdot 10^{7}$	$1.9 \pm 0.1 \cdot 10^{6}$	$1.0 \pm 0.1 \cdot 10^{6}$	$3.1 \pm 0.3 \cdot 10^{5}$
	k_{-1} (s^{-1})	k_{-2} (s^{-1})	k_{-3} (s^{-1})	k_{-4} (s^{-1})
Cd(II)-en	$1.1 \pm 0.1 \cdot 10^{4}$	$2.1 \pm 0.1 \cdot 10^{5}$	$3.2 \pm 0.1 \cdot 10^{6}$	
Cd(II)-nme	$2.1 \pm 0.1 \cdot 10^{6}$	$8.4 \pm 0.1 \cdot 10^{7}$	$8.4 \pm 0.1 \cdot 10^{8}$	$3.7 \pm 0.1 \cdot 10^{9}$
Ni(II)-en	0.26 ± 0.1	2.1 ± 0.3	31 ± 4	
Ni(II)-nme	$1.2 \pm 0.1 \cdot 10^{4}$	$4.8 \pm 0.6 \cdot 10^{4}$	$1.6 \pm 0.1 \cdot 10^{5}$	$6.1 \pm 0.4 \cdot 10^{5}$

Table S 8: Stability constants (pK_i) between different metal-ligand coordination states as evaluated from the ratio of the corresponding formation and dissociation rate constants reported in Supplementary Table 7 exploiting the principle of microscopic reversibility. Stability constants obtained from the free energy profiles are in parentheses.

Ligand conc.	pK_1	pK_2	pK_3	pK_4
Ni(II)-en	7.0<br>(7.1)	5.0<br>(5.5)	2.5<br>(2.9)	
Cd(II)-en	6.1<br>(5.1)	3.8<br>(3.9)	1.55<br>(1.9)	
Ni(II)-nme	2.9<br>(2.2)	1.6<br>(1.5)	0.8<br>(0.6)	-0.3<br>(-0.8)
Cd(II)-nme	3.7<br>(2.5)	2.2<br>(2.0)	0.9<br>(1.0)	-0.4<br>(0.1)

Table S 9: Average number of hydrogen bonds formed between ethylenediamine, putrescine, and methylamine amino groups and water evaluated in the unbound and bound states, considering also the singly-bound (i.e., open ring configuration) and the fully-bound (i.e., closed ring configuration) states. The hydrogen bonds formed by the individual amino groups with water acting as an acceptor or as a donor are reported separately. In the case of en and put, N1 identifies the bounded amino group in the open ring conformation.

En state	N1 donor	N2 donor	N1 acceptor	N2 acceptor
Unbound	0.11	0.11	0.40	0.40
Open ring	0.14	0.13	0.0	0.40
Closed ring	0.18	0.18	0.0	0.0
Put state	N1 donor	N2 donor	N1 acceptor	N2 acceptor
Unbound	0.10	0.10	0.47	0.47
Open ring	0.15	0.10	0.0	0.49
Closed ring	0.20	0.20	0.0	0.0
Nme state	N donor		N acceptor	
Unbound	0.10		0.45	
Bound	0.18		0.0	

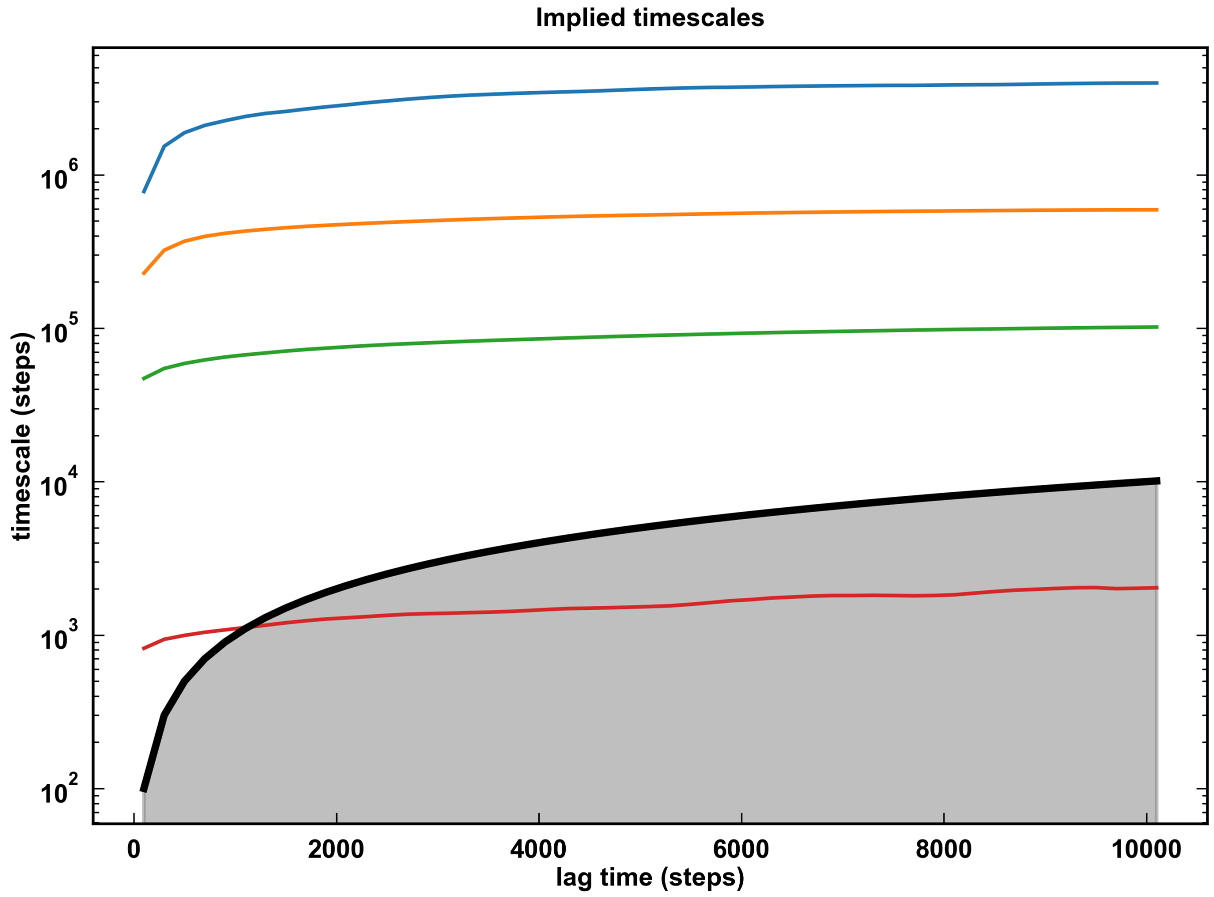

Figure S 1: Implied timescales plot for the 4 slowest eigenvalues associated to the Markov state model of the Cd(II)-en system. After 600 steps (60 ps) the three slowest implied timescales are nicely approximated even for longer lagtimes.

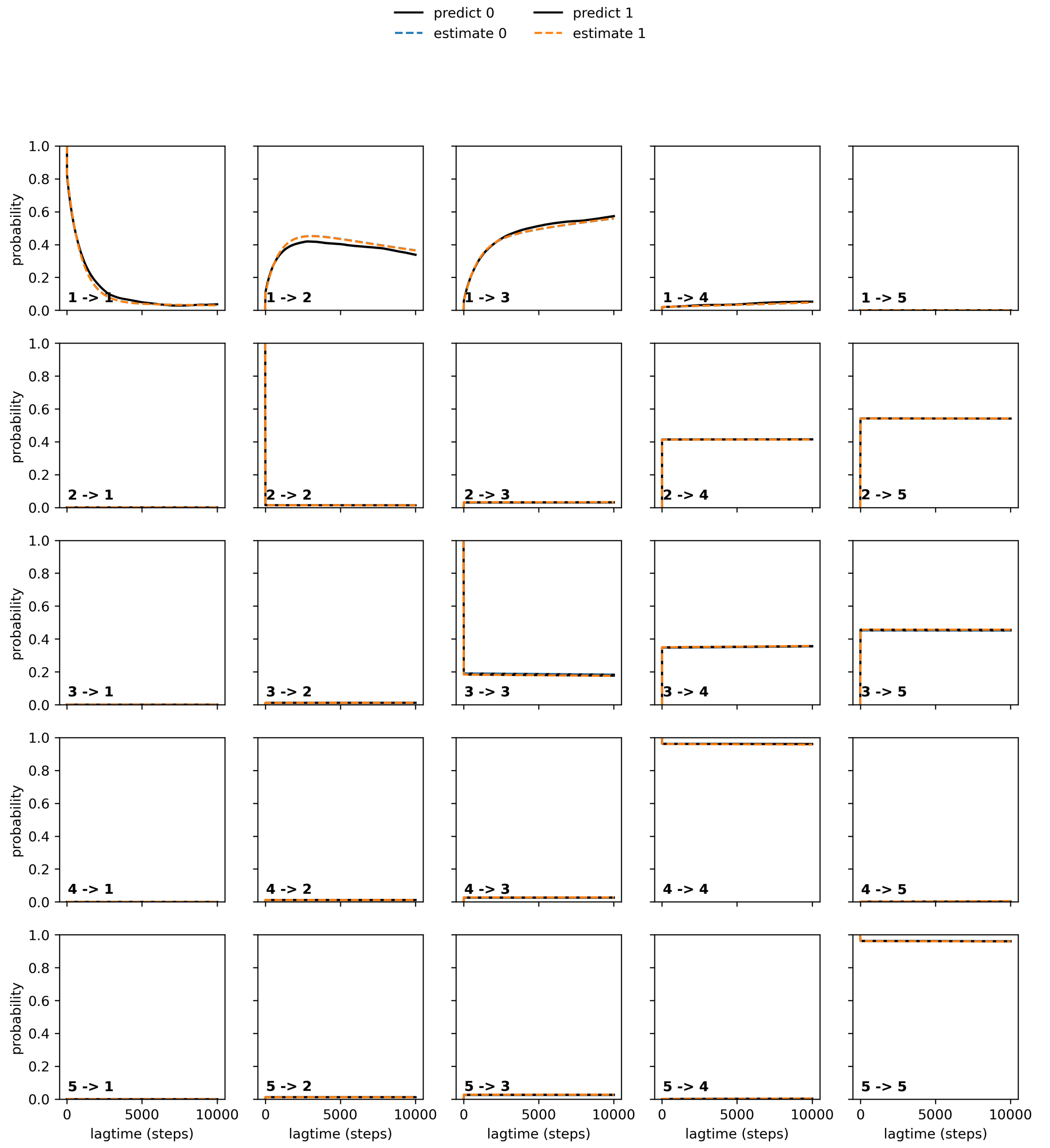

Figure S 2: Chapman-Kolmogorov test of the Cd(II)-en Markov state model. The dynamics between the five metastable states are considered Markovian and are well-reproduced using a lagtime of 60 ps.

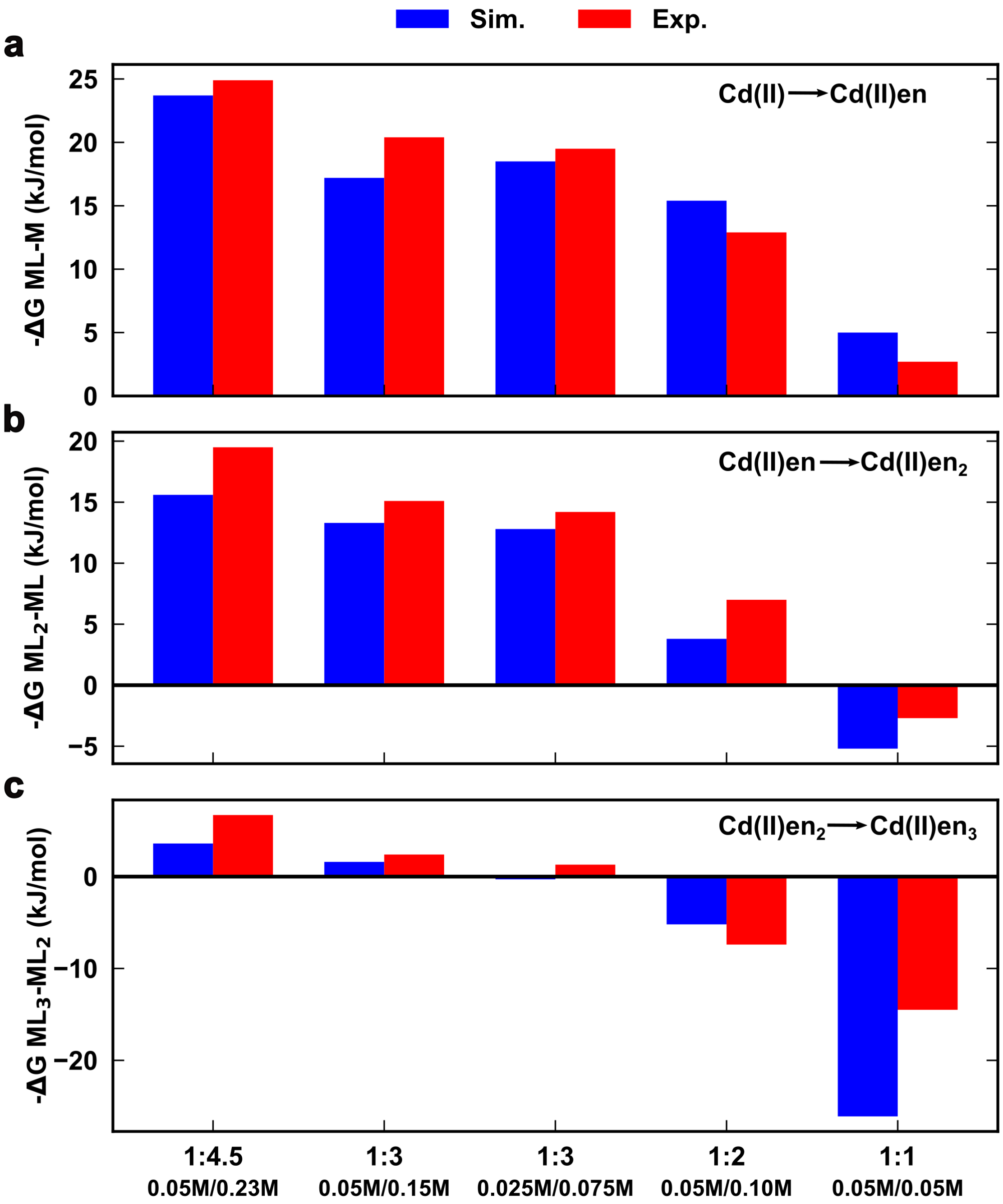

Figure S 3: (a) Relative stability between fully solvated Cd(II) and Cd(en) complex, ΔG^{ML-M}, (b) between Cd(en) and Cd(en)$_2$, ΔG^{ML_2-ML}, and (c) between Cd(en)$_2$ and Cd(en)$_3$, $\Delta G^{ML_3-ML_2}$. Results are shown at different concentrations. In blue the computed results and in red the experimental measured ones.

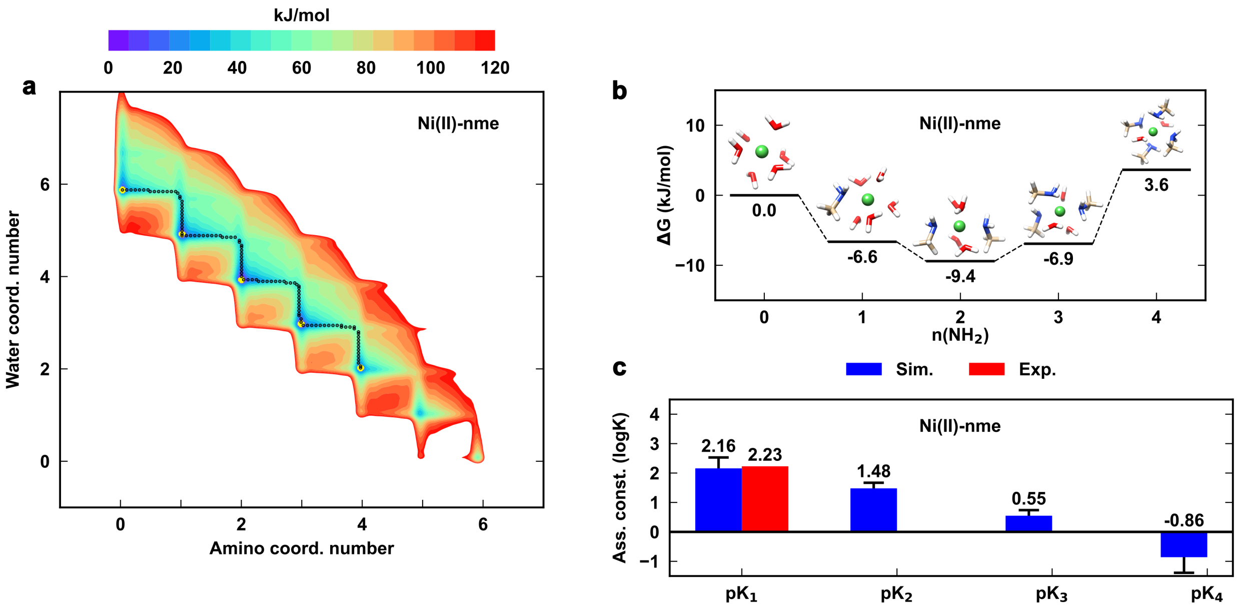

Figure S 4: (a) 2D free energy map of the Ni(II)-nme complex equilibrium in aqueous solution (Ni(II): 0.10M, en: 0.60M), as a function of water and amino coordination number, showing all possible metal coordination states. Yellow points indicate the ML, ML_2, ML_3 and ML_4 configurations. The dotted black line is the minimum free energy pathway. Note that the profile depends on the given concentration. (b) Relative stability of the main Ni(II)-nme complex configurations with respect to the free metal ion. Estimated errors is 2 kJ/mol. (c) Computed and experimental association constants (pK_i) of the ML, ML_2, ML_3 and ML_4 complex species for the Ni(II)-nme system. Estimated errors are reported in Supplementary Table 6.

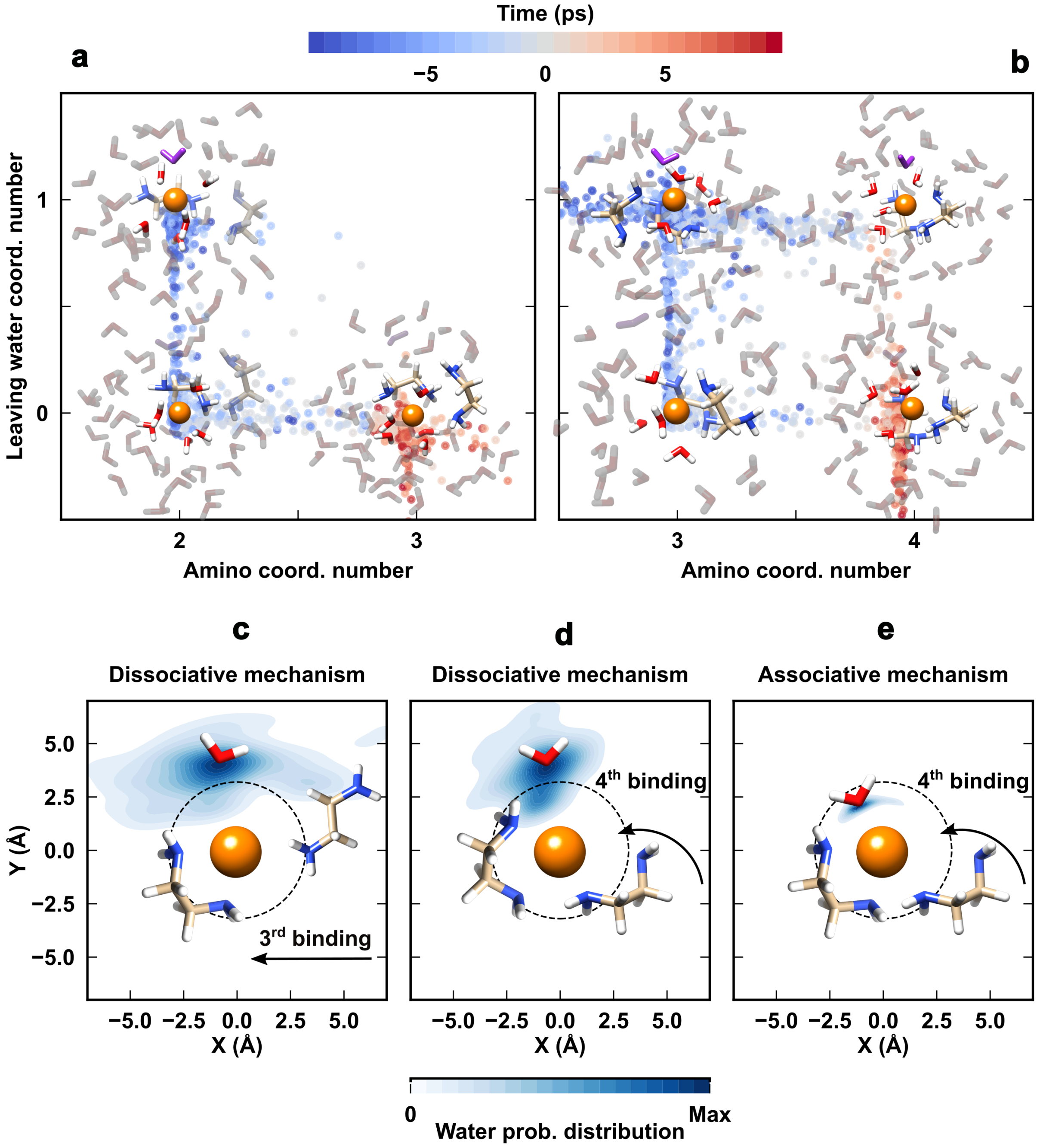

Figure S 5: Evolution of amino and leaving water coordination number in the (a) third and (b) fourth binding event of Cd(II)en_2 formation. The leaving water is represented in purple. Probability distribution of the leaving water in the time interval from 5 ps before to 5 ps after the (c) third dissociative binding, (d) fourth dissociative, and (e) fourth associative binding event of Cd(II)en_2 formation. The black dotted circles represent the Cd(II) first solvation shell.

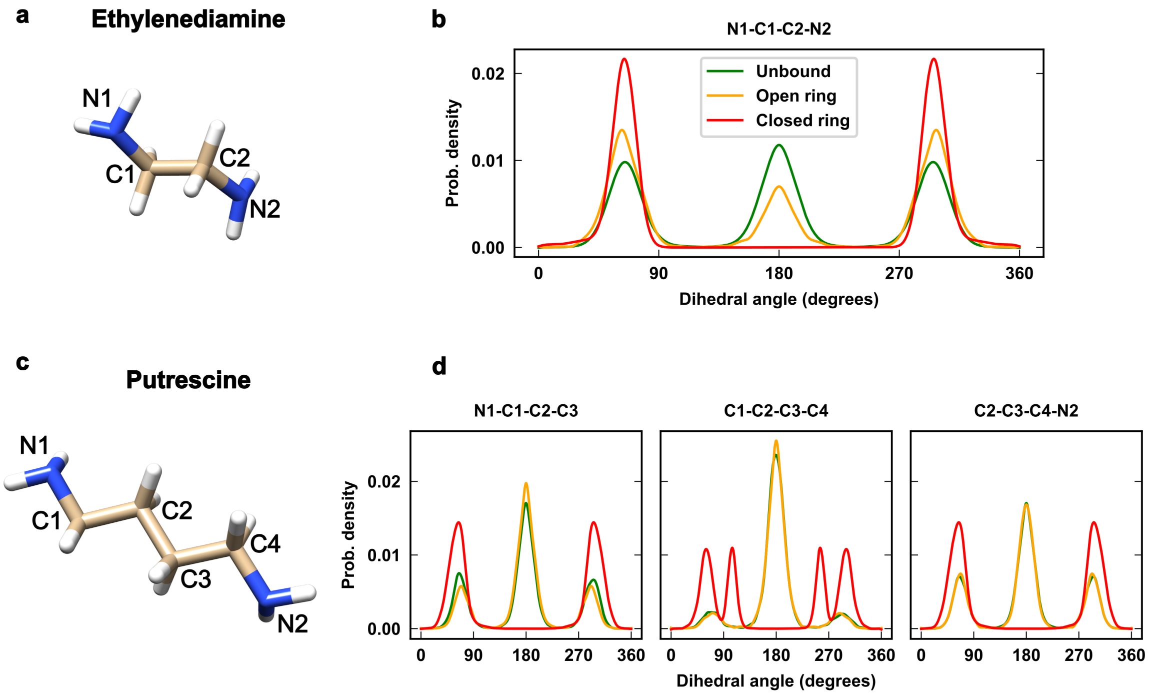

Figure S 6: (a) Structure and atomic labels of the ethylenediamine ligand. (b) Probability distribution of the ethylenediamine dihedral angle in the unbound state (green line), open ring configuration (yellow line), and closed ring configuration (red line). (c) Structure and atomic labels of the putrescine ligand. (d) Probability distribution of the three putrescine dihedral angles in the unbound state (green line), open ring configuration (yellow line), and closed ring configuration (red line). Note that N1 is the amino group bonded to the metal in the open ring configuration.

References

(1) Spike, C. G.; Parry, R. W. Thermodynamics of Chelation. I. The Statistical Factor in Chelate Ring Formation1. *Journal of the American Chemical Society* **1953**, *75*, 2726–2729.

(2) Sengupta, A.; Seitz, A.; Merz, K. M. J. Simulating the Chelate Effect. *Journal of the American Chemical Society* **2018**, *140*, 15166–15169, PMID: 30381949.

(3) Paoletti, P. Formation of metal complexes with ethylenediamine: a critical survey of equilibrium constants, enthalpy and entropy values. *Pure and Applied Chemistry* **1984**, *56*, 491–522.

(4) Pettit, L. D.; Powell, K. The IUPAC stability constants database. *Chem. Int* **2006**, *56*, 14–15.

(5) Taylor, R. W.; Stepien, H. K.; Rorabacher, D. B. Kinetics of aquonickel(II) ion reacting with ethylenediamine. Evidence of the internal conjugate base effect and intramolecular hydrogen bonding. *Inorganic Chemistry* **1974**, *13*, 1282–1289.

(6) Rorabacher, D.; Melendez-Cepeda, C. Steric effects on the kinetics and equilibriums of nickel (II)-alkylamine reactions in aqueous solution. *Journal of the American Chemical Society* **1971**, *93*, 6071–6076.
